# Outcome of Intermittent Thoracentesis versus Pigtail Catheter Drainage for Hepatic Hydrothorax

**DOI:** 10.3390/jcm11237221

**Published:** 2022-12-05

**Authors:** Seul-Ki Han, Seong-Hee Kang, Moon-Young Kim, Seong-Kyun Na, Taehyung Kim, Minjong Lee, Baek-Gyu Jun, Tae-Suk Kim, Dae-Hee Choi, Ki-Tae Suk, Young-Don Kim, Gab-Jin Cheon, Hyung-Joon Yim, Dong-Joon Kim, Soon-Koo Baik

**Affiliations:** 1 Department of Internal Medicine, Yonsei University Wonju College of Medicine, Wonju 26426, Republic of Korea; 2 Regenerative Medicine Research Center, Yonsei University Wonju College of Medicine, Wonju 26426, Republic of Korea; 3 Department of Internal Medicine, Inje University College of Medicine, Seoul 01757, Republic of Korea; 4 Department of Internal Medicine, Korea University College of Medicine, Seoul 01757, Republic of Korea; 5 School of Medicine, Ewha Womans University, Seoul 07985, Republic of Korea; 6 Department of Internal Medicine, Kangwon National University Hospital, Chuncheon 24289, Republic of Korea; 7 Department of Internal Medicine, Hallym University College of Medicine, Chuncheon 24289, Republic of Korea; 8 Department of Internal Medicine, University of Ulsan College of Medicine, Gangneung 25440, Republic of Korea

**Keywords:** hepatic hydrothorax, pigtail catheter, intermittent thoracentesis, liver cirrhosis

## Abstract

**Background/Aims:** The management of hepatic hydrothorax (HH) remains a challenging clinical scenario with suboptimal options. We investigated the effect and safety of pigtail catheter drainage compared to intermittent thoracentesis. **Methods:** This multicenter, retrospective study included 164 cirrhotic patients with recurrent pleural effusion from March 2012 to June 2017. Patients with neoplasms, cardiopulmonary disease, and infectious conditions were excluded. We compared the clinical outcomes of pigtail catheter drainage versus thoracentesis for variables including complications related to procedures, overall survival, and re-admission rates. **Results:** A total of 164 patients were divided into pigtail catheter (*n* = 115) and thoracentesis (*n* = 49) groups. During the follow-up period of 6.93 months after discharge, 98 patients died (pigtail; *n* = 47 vs. thoracentesis; *n* = 51). The overall survival (*p* = 0.61) and 30-day mortality (*p* = 0.77) rates were similar between the pigtail catheter and thoracentesis groups. Only MELD scores were associated with overall survival (adjusted HR, 1.08; *p* < 0.01) in patients with HH. Spontaneous pleurodesis occurred in 59 patients (51.3%) in the pigtail catheter group. Re-admission rates did not differ between the pigtail catheter and thoracentesis groups (13.2% vs 19.6% *p* = 0.7). A total of five complications occurred, including four total cases of bleeding (one patient in the pigtail catheter group and three in the thoracentesis group) and one case of empyema in the pigtail catheter group. **Conclusions:** Pigtail catheter drainage is not inferior to that of intermittent thoracentesis for the management of HH, proving it may be an effective and safe clinical option.

## 1. Introduction

Hepatic hydrothorax (HH) is defined as pleural effusion associated with liver cirrhosis and/or portal hypertension [1,2]. Several factors are known to contribute to the development of effusion; the most accepted mechanism of which is the direct delivery of ascites fluid from the peritoneal cavity to the pleural cavity through microscopic congenital diaphragmatic defects. Although less common, it can also develop in up to 15% of patients with portal hypertension [2,3,4,5,6]. HH carries a high mortality with up to 25% of patients dying within one year of diagnosis [1,2].

Standard management for HH is diuresis along with fluid and salt restriction. When HH becomes refractory to standard therapy, patients often require invasive procedures including intermittent thoracentesis, chest tube insertion for symptom management, transjugular intrahepatic portosystemic shunt (TIPS), and liver transplantation. Unfortunately, many patients with HH have contraindications to TIPS such as hepatic encephalopathy and hyperbilirubinemia.

According to current practice guidelines from the American Association for the Study of Liver Diseases, the placement of chest tubes for the management of HH is considered contraindicated because of extremely high morbidity and mortality rates of between 80 and 100% [2,3,7,8,9,10]. Although intermittent thoracentesis can provide rapid relief of symptoms, it is a temporary fix and needs to be repeated. As a result, complications such as re-expansion pulmonary edema, pneumothorax, bleeding, and empyema increase with the repeated procedures [2,3,7,11].

Small-bore drainage was first found to be feasible in treating malignant effusions [12]. The pigtail catheter is a small-bore drainage catheter that is used to prevent accidental dislodgment. Pigtail catheterization can be performed easily and quickly under ultrasound guidance at the patient’s bedside. Mohamed at al. reported that pigtail catheter drainage was successful in managing HH with low complication levels [13]. We aimed to evaluate the efficacy and safety of pigtail catheter insertion compared to intermittent thoracentesis in HH management.

## 2. Methods

### 2.1. Patients

A multicenter, retrospective analysis was performed on all patients with a diagnosis of cirrhosis who were admitted to five tertiary university hospitals for the first time with recurrent pleural effusion that was refractory to standard therapy, between January 2012 and June 2017. Inclusion criteria were the presence of bilateral or unilateral transudate fluid that met Light’s criteria (pleural fluid-to-serum protein ratio < 0.5, pleural fluid lactic dehydrogenase [LDH] < 200 IU, and pleural fluid-to-serum LDH ratio < 0.6), with cirrhosis. Exclusion criteria included patients with a pleural effusion due to a condition other than HH or those who had a diagnosis of empyema, tuberculosis pleurisy, parapneumonic effusion, inflammatory pleuritis, as well as hepatocellular carcinoma, other neoplasms, and congestive heart failure. We excluded the patients who need repeated large volume paracentesis at least once a week. At admission, all patients had paracentesis for abdominal distension fewer than one time. This study protocol was approved by the Institutional Review Boards (CR318098) and the requirement for informed consent was waived. This study was performed in accordance with the Declaration of Helsinki.

Each patient was followed-up on an individual basis with an electronic medical recording system. If the date of death or catheter removal was not available, data were censored on the date that records were received from the participating institution. The index date of follow-up is the date of admission for the first time with recurrent pleural effusion. Time from index date to the study end date in the participating center or time to death was used to estimate survival. The choice of either pigtail catheter drainage or thoracentesis depended on the physician’s decisions. From the time patients were included, to mortality or their last follow-up, additional data were collected on procedure-related complications, re-admission rates, and liver related mortality.

### 2.2. Procedures

Intermittent thoracentesis was done by ultrasonographic guidance. All thoracentesis drainage used a vinca catheter (16 gauge) under local anesthesia (lidocaine 1%). The volume of fluid to be drained varied depending on the level of effusion. In general, drainage of less than 1500 mL was performed to reduce the occurrence of re-expansion pulmonary edema, and the drainage procedures were repeated as necessary based on symptom recurrence or pleural fluid re-accumulation.

Pigtail catheterization used a size 8F pigtail catheter with trocar. Needles were inserted just above the top of the rib to avoid injury of the intercostal bundle, under local anesthesia (lidocaine 1%). Insertion was done by advancing the trocar and catheter until reaching the pleural cavity, then gradually withdrawing the trocar while simultaneously introducing the catheter. The catheter was then connected to a collecting bag via a triple-way valve and placed on water–seal for drainage. Pigtail catheters were removed when drainage was less than 100 mL daily for three successive days, or at any time after the procedure if there were signs of infection or catheter malfunction. All catheterized patients received the standard therapy for HH according to the guidelines (e.g., diuretics and salt restriction). The procedure was performed depending on the physician’s proficiencies, preference, and equipment of the institution. In general, one out of five institutions performed predominantly pigtail catheter drainage and the remaining four institutions mainly performed thoracentesis for HH. After a single drainage was done, we administered albumin replacement of 20 g for the prevention of hypovolemia.

### 2.3. Outcomes

The primary outcome was establishing the safety and efficacy of pigtail catheter drainage and intermittent thoracentesis. Standard definitions and medical judgment were used for identification of adverse events, including empyema, bleeding, pain, pneumothorax, and skin infection associated with the procedures. Efficacy was defined as time to spontaneous pleurodesis and the liver transplant-free survival period. Re-admission rates, 30-day mortality, the degree of hypoalbuminemia, electrolyte imbalances after procedure, and the occurrence of an acute kidney injury or hepatorenal syndrome were analyzed and compared between patients who underwent intermittent thoracentesis and pigtail catheter insertion.

### 2.4. Definitions

We considered the case to be spontaneous pleurodesis if HH did not recur and did not require additional procedures, and the patient was maintained on diuretic treatment without re-admission.

Catheter site cellulitis was defined as erythema anywhere along the tunneled catheter insertion site, with or without expressible purulent material, without evidence of empyema. Bleeding events included minor and major bleeding that needed transfusions.

### 2.5. Statistical Analysis

We analyzed the pigtail catheter drainage and thoracentesis groups separately using the Chi-square test and the independent *t*-test to evaluate the differences in clinical variables. We calculated the cumulative rates of overall survival and re-admission using the Kaplan–Meier method and excluded the patients who were lost to follow-up from the analysis. We performed the log-rank test to compare the differences between the groups. Predictors of mortality were calculated by univariate and multivariate Cox regression analyses. *p*-values < 0.05 were considered significant. The statistical analyses were performed using SPSS for Windows, version 23.0 (SPSS Inc., Chicago, IL, USA).

## 3. Results

**Figure 1:**
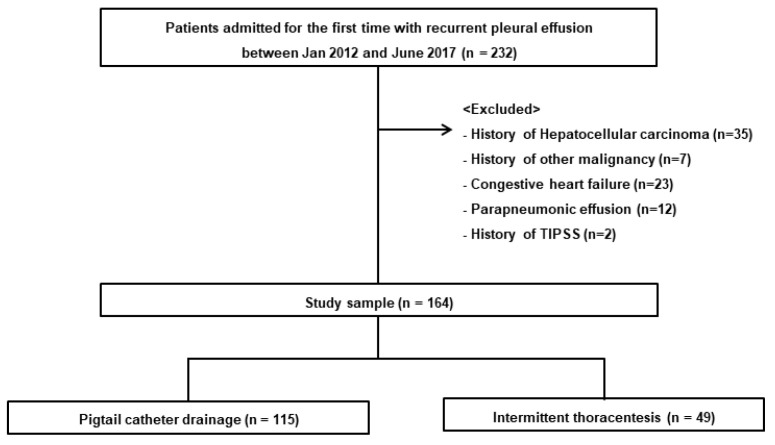
Patient flow diagram.

A total of 164 patients were retrospectively analyzed and divided into pigtail catheter (*n* = 115) and thoracentesis (*n* = 49) groups (Figure 1). The median follow-up period was 6.93 months (IQR 0.83–22.6). There were no significant differences in age, sex, etiology of cirrhosis, baseline creatinine or MELD scores between the pigtail catheter and thoracentesis groups (Table 1).

**Figure 2:**
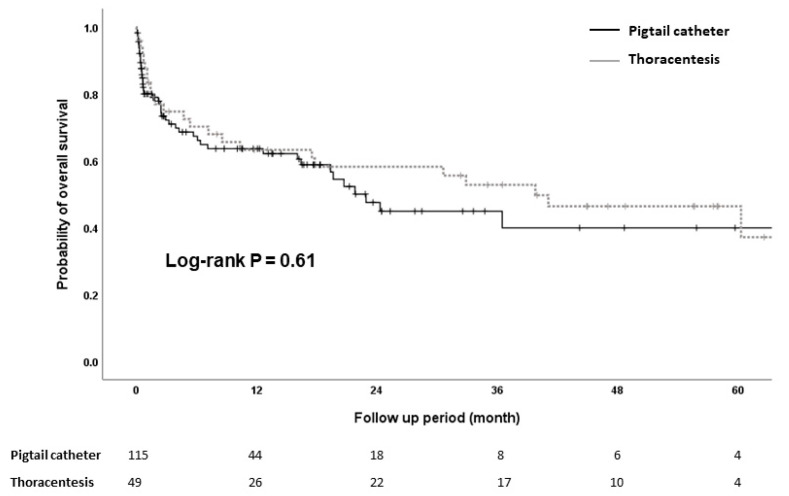
Kaplan–Meier survival analysis for cirrhotic patients with hepatic hydrothorax requiring drainage.

During the follow-up period after discharge, 98 patients died: 47 patients in the pigtail catheter group and 51 patients in thoracentesis group. The cumulative probabilities of survival at 12, 24, 36, and 60 months were 63.6%, 44.9%, 39.9%, and 39.9% in the pigtail catheter group and 63.3%, 58.2%, 52.8%, and 37.1% in thoracentesis group, respectively (Figure 2). Univariate analysis showed that the association of type of management of HH with survival was not statistically significant (HR, 0.88; 95% CI, 0.53–1.45; *p* = 0.62). Multivariate analysis showed that only MELD scores were associated with overall survival (adjusted HR [aHR], 1.08; 95% CI, 1.03–1.13; *p* < 0.01) in patients with HH (Table 2).

The cumulative incidence of 30-day survival was similar between the pigtail catheter group and thoracentesis group (*p* = 0.77). The overall 30-day mortality for patients treated with pigtail catheter drainage versus intermittent thoracentesis was 20.0% and 12.4%, respectively.

### 3.1. Hospitalization Period

The mean hospitalization period of the pigtail catheter group was not different from that of the thoracentesis group (19.6 ± 17.9 vs. 15.7 ± 20.1 days; *p* = 0.23). In the pigtail catheterization group, during hospitalization, the average catheterization period was 8.4 ± 7.9 days, and in the follow-up period, there were 59 cases of spontaneous pleurodesis (51.3%). In the thoracentesis group, repeat thoracentesis was required, and an average of 1.5 ± 0.93 procedures were performed per person.

### 3.2. Re-Admission Rate

**Figure 3:**
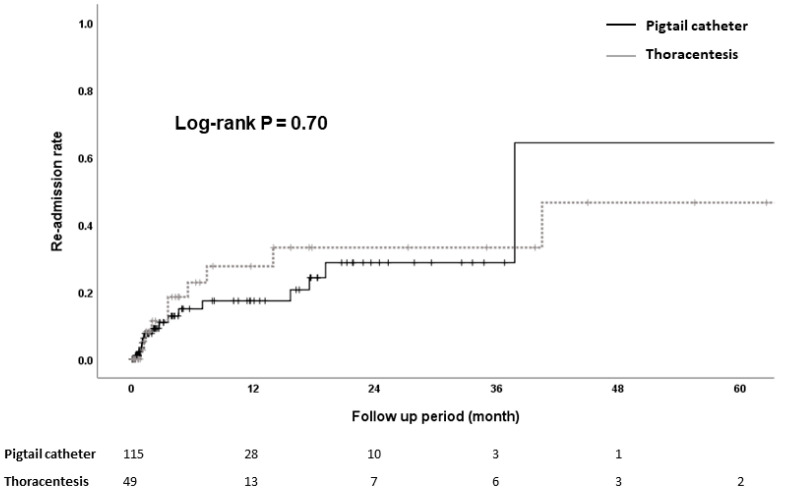
Re-admission rate after the procedure because of recurrence of hydrothorax.

Twenty-five patients were readmitted because of recurrence of HH during the follow-up period. Re-admission rates were similar between the pigtail catheter group and thoracentesis group (13.2% vs. 19.6%, *p* = 0.70) (Figure 3). The median time for re-admission was 82 days [IQR, 31–470] in the pigtail catheter group and 108 days [IQR, 40.7–212.5] in the thoracentesis group.

### 3.3. Complications

There were no occurrences of immediate complications related to the procedures (pneumothorax, loculation, pain requiring removal or skin infection) (Table 3). There was one case of empyema in the pigtail group. The patient was catheterized for six days, hospitalized for twenty-eight days, and discharged after conservative management, as the infection was successfully treated with antibiotics and drainage. However, post-procedure bleeding was present in four patients: one patient in the pigtail catheter group and three patients in the thoracentesis group. In the thoracentesis group, two patients expired during hospitalization. One of them died of liver failure five days after hospitalization, while the other one experienced minor bleeding, but it was well-controlled. On day twenty-six, however, the patient expired because of spontaneous bacterial peritonitis. Of the three patients with bleeding, the last patient was discharged. In the pigtail catheter group, the patient with post-procedure bleeding was treated with conservative management, including a transfusion, and discharged.

### 3.4. Changes in Albumin and Creatinine Post-Procedure

To evaluate catheter drainage complications, changes in albumin and creatinine levels were analyzed seven and fourteen days after the procedures, respectively. The mean baseline serum albumin level was 2.72 g/dL (range, 1.2–3.9 g/dL), with a mean follow-up level of 2.74 g/dL (range, 1.8–4.0 g/dL) and 2.87 g/dL (range, 1.7–3.8 g/dL) seven and fourteen days post-procedure, respectively. After seven days, albumin demonstrated a mean increase of 0.06 g/dL in the pigtail catheter group and of 1.48 g/dL in the thoracentesis group. However, after fourteen days, albumin demonstrated a mean decrease of −0.54 g/dL in the pigtail catheters group, but an increase of 0.81 in the thoracentesis group, which reached statistical significance (*p* < 0.01).

The mean baseline serum creatinine level was 1.29 g/dL (range, 0.2–9.0 g/dL), with a mean follow-up level of 1.13 g/dL (range, 0.2–4.78 g/dL) and 1.17 g/dL (range, 0.23–6.01 g/dL) seven and fourteen days post-procedures, respectively. There were no statistically significant changes in creatinine levels between the pigtail catheter and the thoracentesis group on day seven or fourteen.

## 4. Discussion

We assessed the use of pigtail catheterization compared to thoracentesis as a safe and practical method for treatment of HH. This is the first study to compare pigtail catheter insertion and intermittent thoracentesis in HH management. Thoracentesis is a relatively easy method that is useful in clinical practice. However, repeated thoracentesis has recently been shown to be associated with a significantly higher complication rate and impaired quality of life in patients with HH who require frequent visits to the clinic. In that regard, Eldin et al. devised a new technique by using small-bore pigtail catheter for the management of HH and conducted a prospective study with 60 patients [13]. These results demonstrated that severe complications such as infection and pneumothorax were absent, though 12 (20%) patients had pain at the site of insertion. Pleurodesis was performed on 38 (63.3%) patients with no recurrence of fluid within three months. However, there was no study comparing the effectiveness and safety of the novel pigtail catheter drainage to the more established thoracentesis for HH treatment. Thus, our study compared both methods directly and showed that the mortality rates of patients treated with pigtail catheter drainage did not differ from those treated with thoracentesis. Moreover, the complications of pigtail catheter drainage were milder, though the overall incidence was comparable to that of thoracentesis group.

Tunneled indwelling pleural catheter (IPC, also known as PleurX or the Denver catheter) was first introduced in 1997 and initially intended for palliative outpatient therapy of recurrent malignant pleural effusions [14], though it is currently emerging as an alternative option to chest tube insertion [8,9,10,15,16]. In a recent meta-analysis study that including 10 studies involving a total of 269 hepatic hydrothorax cases, 47% of patients with IPC achieved spontaneous pleurodesis. This result is similar to the rate in our analysis. The lower complications rate for IPC can be an alternative therapeutic option for hepatic hydrothorax [17]. However, the data regarding IPCs for HH have been limited to case reports and small studies. Moreover, the use of IPC is not yet available in many countries, depending on the region and health system. Conversely, pigtail catheter is a drainage method that has been used for a long time for other diseases and remains a viable treatment option in institutions with limited access to IPC.

There is no clearly favored method for symptomatic relief in cirrhotic patients with pleural effusion because of the heterogeneity in results among previous studies. Hung et al. used Taiwan’s nationwide population-based dataset for evaluation of mortality rates associated with the drainage methods (catheter drainage versus thoracentesis) [17]. This comparison study showed, contrary to our results, that catheter drainage was associated with a higher 30-day mortality compared with thoracentesis (23.5 versus 18.6%; *p* < 0.001). The above-mentioned study did not excluded patients treated with chest tubes and lacked detailed clinical data because it was a nationwide population-based study. Moreover, the etiology of the pleural effusions could not be evaluated extensively in this study because fluid analysis data were lacking. Because of these limitations, it is difficult to assume that pigtail drainage and thoracentesis were directly compared, and to accept the result that pigtail drainage is associated with higher mortality compared to thoracentesis.

In the present study, complications were few and included empyema in one (0.8%) patient, and bleeding in only one (0.8%) patient. Pneumothorax did not occur in any of our patients. Previous studies using pigtail catheters for pleural drainage revealed few complications. Only 5% of pigtail catheters performed in the study by Roberts et al. had serious complications like pneumothorax, hemothorax, and hepatic perforation [18]. Another study for treatment of malignant pleural effusions reported that pneumothorax occurred in four out of fifteen patients and spontaneously resolved [19]. Therefore, these data suggest that pigtail catheter drainage is a safe and possible bridge treatment before TIPS or liver transplantation.

One additional concern with pigtail drainage in patients with HH is that repeated drainages may cause nutritional or electrolyte disruption in these individuals, and it is unclear whether we should be replacing albumin intravenously as recommended with large volume paracentesis. There is no confirmed data on the effect of albumin infusion in conjunction with thoracentesis [1]. We examined the effect of pleural effusion drainage on nutritional factors such as serum albumin and on renal function, finding a small downward trend seven days after pigtail drainage. However, the absolute loss of albumin was small and of questionable clinical significance. In terms of renal function, there was no increase of serum creatinine levels in either pigtail catheterization or thoracentesis. However, it should be noted that eight (7%) pigtail catheter patients and six (11.7%) thoracentesis patients demonstrated hepatorenal syndrome (HRS). It is difficult to determine if HRS is entirely a complication of the drainage, as patients with HH are prone to HRS, and HRS may spontaneously occur in 8% of patients with MELD scores of about 10 at the one year mark [20,21]. Generally, when MELD scores approach 18, nearly 40% of patients develop HRS within a year. Considering the mean MELD score was 19 in this study, the incidence of hepatorenal in 7–11% is acceptable. However, patients who required pleural effusion drainage were on regular oral diuretic therapy for volume control and developed acute kidney injury. As such, we recommend that patients who have either pigtail insertion or repetitive thoracentesis have their serum electrolytes and renal function measured regularly.

This study has some limitations. Firstly, this was a retrospective study with a small sample size. Therefore, we did not have reliable information regarding the using of diuretics, dosage, and treatment compliance. However, our cohort represents real-world insight into patients with established cirrhosis and HH. In addition, this study has limitations on distinguishing whether HH is an event that has been repeated or is the first event. Finally, the major limitation of our analysis was that individual physicians at independent locations decided the treatment strategy. Therefore, there was potential for selection bias.

We present multicenter, retrospective data comparing the mortalities associated with different drainage methods for pleural effusion in cirrhotic patients with HH. This study suggests that the pigtail catheter drainage may be an effective procedure that results in spontaneous pleurodesis for 51% of patients. The complications of pigtail catheter drainage are overall comparable to that of thoracentesis. Although IPC is being proposed as an effective treatment for HH, we believe that pigtail catheter drainage can be a safe alternative where IPC use is still limited. However, future controlled studies are also needed to better assess the effects of this approach regarding infection risk, nutritional status, and use in transplant patients.

## Figures and Tables

**Table 1:** Baseline characteristics of patients.

	Pigtail Catheter (*n* = 115)	Intermittent Thoracentesis (*n* = 49)	*p*-Value
Age (years)	62.69 ± 12.72	59.71 ± 12.41	0.17
Sex, Male, *n* (%)	87 (75.6)	35 (71.4)	0.57
Etiology, *n* (%)			0.64
HBV	20 (17.4)	10 (20.4)	
HCV	11 (9.6)	5 (10.2)	
Alcohol	78 (67.8)	30 (61.2)	
Others	6 (5.2)	4 (8.2)	
ALT	43.27 ± 88.94	33.13 ± 27.93	0.43
AST	107.61 ± 181.98	86.27 ± 140.84	0.46
Albumin (g/dL)	2.74 ± 0.50	2.67 ± 0.51	0.42
Total bilirubin (mg/dL)	4.77 ± 6.36	5.89 ± 7.17	0.32
PT (INR)	1.69 ± 0.55	1.70 ± 0.54	0.93
Creatinine	1.40 ± 1.27	1.04 ± 0.48	0.08
Child Pugh Stage, *n* (%)			0.06
*A*	14 (12.2)	2 (4.1)	
*B*	56 (48.7)	19 (38.8)	
*C*	45 (39.1)	28 (57.1)	
MELD	19.78 ± 7.86	19.17 ± 7.64	0.64

Abbreviations: HBV, hepatitis B virus; HCV, hepatitis C virus; MELD; Model for End Stage Liver Disease; PT, prothrombin time.

**Table 2:** Variables independently associated with overall survival.

Variables	Univariate Analysis			Multivariate Analysis		
	HR	95% CI	*p*-Value	HR	95% CI	*p*-Value
Sex (male)	0.69	0.48–1.63	0.69	0.75	0.35–1.59	0.45
Age (year)	0.99	0.97–1.01	0.53	1.00	0.98–1.03	0.52
Etiology of cirrhosis						
*Viral*	Ref					
*Alcohol*	1.64	0.93–2.91	0.08			
*Others*	1.37	0.50–3.76	0.53			
Albumin (g/dL)	0.68	0.42–1.10	0.12			
Creatinine (mg/dL)	1.21	1.06–1.39	<0.01			
PT (INR)	0.98	0.96–1.00	0.23			
Total Bilirubin (mg/dL)	1.04	1.01–1.08	0.01			
Child Pugh Stage						
*A*	Ref			Ref		
*B*	2.66	0.62–11.35	0.18	1.39	0.30–6.32	0.67
*C*	7.64	1.85–31.58	<0.01	3.19	0.64–15.80	0.15
MELD score	1.09	1.05–1.12	<0.01	1.08	1.03–1.13	<0.01
Type of management for HH	0.88	0.53–1.45	0.62	0.40	0.15–1.06	0.07
*Intermittent thoracentesis*	Ref			Ref		
*Pigtail catheter*	0.88	0.53–1.45	0.62	0.40	0.15–1.06	0.07

Abbreviations: CI, confidence interval; HH, hepatic hydrothorax; HR, hazard ratio; MELD, Model for End Stage Liver Disease; PT, prothrombin time; Ref, reference.

**Table 3:** Outcomes of interest.

	Pigtail Catheters (*n* = 115)	Intermittent Thoracentesis (*n* = 49)	*p*-Value
Clinical outcomes			
*Spontaneous pleurodesis, n (%)*	59 (51.3)		
*Catheter maintenance period (day)*	8.4 ± 7.9		
*Median number of thoracentesis at hospitalization*		1.0 [1.0, 2.0]	
*Total volume of drainage* (mL)	2610 [1000,5795]		
*Hospitalization period (days)*	18.9 ± 16.0	13.3 ± 13.4	0.04
*Readmission d/t hepatic hydrothorax, n (%)*	15 (13.2)	10 (19.6)	0.74
*Hepatorenal syndrome, n (%)*	8 (7.0)	6 (11.7)	0.32
Procedure related complication			0.11
*Loculation*	0	0	
*Empyema*	1	0	
*Bleeding*	1	3	
*Pain requiring removal*	0	0	
*Pneumothorax*	0	0	
*Cellulitis*	0	0	

Hepatorenal syndrome (HRS) developed in eight cases in the pigtail group and in six cases in the thoracentesis group, during hospitalization.

## Data Availability

Not applicable.

## Abbreviations

ALT: alanine aminotransferase; AST, aspartate aminotransferase; CTP, child-turcotte-pugh; HBV, hepatitis B virus; HCV, hepatitis C virus; HH, hepatic hydrothorax; HRS, hepatorenal syndrome; INR, international normalized ratio; IPC, Indwelling pleural catheter; LDH, lactic dehydrogenase; MELD, model for end-stage liver disease; TIPS, transjugular intrahepatic portosystemic shunt.
